# ‘There is no such thing as getting sick justly or unjustly’ – a qualitative study of clinicians’ beliefs on the relevance of personal responsibility as a basis for health prioritisation

**DOI:** 10.1186/s12913-020-05364-6

**Published:** 2020-06-03

**Authors:** Gloria Traina, Eli Feiring

**Affiliations:** Department of Health Management and Health Economics, University of Oslo, Post box 1089 Blindern, 0317 Oslo, Norway

**Keywords:** Norway, Personal health responsibility, Clinical priority setting, Clinicians’ beliefs, Vignettes, Conceptually driven thematic analysis

## Abstract

**Background:**

Concerns have been raised regarding the reasonableness of using personal health responsibility as a principle or criterion for setting priorities in healthcare. While this debate continues, little is known about clinicians’ views on the role of patient responsibility in clinical contexts. This paper contributes to the knowledge on the empirical relevance of personal responsibility for priority setting at the clinical level.

**Methods:**

A qualitative study of Norwegian clinicians (*n* = 15) was designed, using semi-structured interviews with vignettes to elicit beliefs on the relevance of personal responsibility as a basis for health prioritisation. Sampling was undertaken purposefully. The interviews were conducted in three hospital trusts in South-Eastern Norway between May 2018 and February 2019 and were analysed with conceptually driven thematic analysis.

**Results:**

The findings suggest that clinicians endorsed a general principle of personal health responsibility but were reluctant to introduce personal health responsibility as a formal priority setting criterion. Five main objections were cited, relating to avoidability, causality, harshness, intrusiveness, and inequity. Still, both retrospective and prospective attributions of personal responsibility were perceived as relevant in specific clinical settings. The most prominent argument in favour of personal health responsibility was grounded in the idea that holding patients responsible for their conduct would contribute to the efficient use of healthcare resources. Other arguments included fairness to others, desert and autonomy, but such standpoints were controversial and held only marginal relevance.

**Conclusions:**

Our study provides important novel insights into the clinicians’ beliefs about personal health responsibility improving the empirical knowledge concerning its fairness and potential applications to healthcare prioritisation. These findings suggest that although personal health responsibility would be difficult to implement as a steering criterion within the main priority setting framework, there might be clinical contexts where it could figure in prioritisation practices. Additional research on personal health responsibility would benefit from considering the multiple clinical encounters that shape doctor-patient relationships and that create the information basis for eligibility and prioritisation for treatment.

## Background

The debates concerning healthcare priority setting have prompted more explicit decision-making at the macro, meso, and ‘bedside’ levels of health services delivery. There is, however, no overall agreement about the most appropriate ethical principles and criteria to guide priority setting decisions in healthcare [1], and multi-criteria approaches to resource allocation have been suggested as a possible solution [2–4].

In recent years, the proposal that personal health responsibility should be taken into account in setting priorities regarding access to treatment has been widely discussed in the academic literature [5–13].

Empirical knowledge on clinicians’ attitudes towards personal health responsibility as a possible prioritisation criterion is still limited yet growing. Previous studies indicate that considerations about personal health responsibility and lifestyle might be regarded as relevant for clinical decisions on patients’ eligibility and prioritisation for treatment [14–17]. For example, research on priority setting practices for bariatric surgery in the United Kingdom has emphasised that clinicians have used a weight loss target as a criterion for accessing care and required patients to show due diligence by changing their lifestyle before they were offered surgery [18, 19]. Similarly, alcoholic liver disease patients have been subjected to additional protocols (including abstinence requirements and psychiatric evaluations) that affect their status and priority on the waiting list [20]. Studies on disease prestige have revealed that conditions that might be viewed as ‘self-inflicted’, such as hepatocirrosis in alcholic patients, hold low prestige among doctors [21, 22]. Moreover, hepatitis C-positive injection drug users have been shown to receive suboptimal treatment, with some evidence pointing to a reluctance among clinicians to treat patients with ongoing drug addictions [23]. The elegibility of this patient group for treatment often depends on an evaluation of their ability to adhere to and be motivated for therapy [24].

Such prioritisation practices might originate from policies and guidelines at the macro and meso levels which, in some countries, have adopted mechanisms of conditional access to healthcare services based on personal lifestyles or behavioural change [8, 25, 26].

In Norway, the Specialist Healthcare Act affirms that the provision of specialist care shall be regulated by the following three priority setting criteria: the health benefit of the medical intervention, its cost-effectiveness, and the severity of the medical condition [27]. Policy makers in Norway have been reluctant to include personal health responsibility in the set of criteria that regulates healthcare prioritisation, but key health policy documents discussing this criterion suggest that considerations regarding a patient’s lifestyle could be given weight in specific priority setting situations at the clinical level [28]. The Norwegian Directorate of Health is responsible for developing, in collaboration with medical experts, national and clinical guidelines for priority setting based on the three criteria of health benefit, cost-effectiveness and severity. Still, there is variation as to how specifically the three criteria are operationalised in such guidelines. Moreover, the guidelines are not binding for the healthcare provider, and individual circumstances can be taken into account for the prioritisation of single patients by clinicians at the ‘bedside’ [29].

Despite the increased focus on explicit priority setting practices [30, 31], prioritisation decisions at the clinical level can be rather implicit [32]. Given the key role played by clinicians in priority setting decisions at the clinical level and the increasing burden of non-communicable diseases (NCDs) related to lifestyle, it is of great interest to understand how clinicians view the role of personal health responsibility in decisions on eligibility and priority for treatment.

This qualitative study aims at investigating clinicians’ beliefs on the relevance of patient self-responsibility in clinical priority setting. Specifically, we address the following research questions:

1. How do clinicians understand the role of personal health responsibility in priority setting?
2. How do clinicians justify or object to applications of personal health responsibility in priority setting?

## Methods

The study was designed as a qualitative interview study. Data were analysed thematically with the use of a predetermined conceptual framework. Ethical approval was granted by the Norwegian Centre for Research Data (reference number 50172).

### Study setting

The Norwegian healthcare system is national and tax-financed and provides universal access to health and care services. A right to ‘equal access to high-quality healthcare services’, regardless of gender, social and economic background, and geographical location is granted by the Patient and User’s Rights Act [33]. Patients have a right to receive necessary care when there is an expected health benefit from the intervention and when the expected costs are reasonably proportionate to the health benefit. Priorities should be set by three criteria of expected health benefit, severity of the disease and cost-effectiveness of the intervention [34].

Specialised care is delivered through hospital trusts organised under four regional health authorities (RHAs) and available through general practitioner (GP) referral [35]. The Norwegian Ministry of Health and Care Services provides the RHAs with budgets and instructions as to current aims and priorities. Although the RHAs are required by law to not exceed the provided budgets, there has been a tendency of the Parliament to allow increases in levels of funding [35]. After a phase of increased cost control between 2005 and 2014, recent years have seen a new trend of activity growth at hospitals and softer budgets [36]. Still, the government recognises that rising costs related to demographic shifts and innovations in expensive health technologies set higher requirements for efficiency and prioritisation in Norwegian specialist care [37].

### Sampling and recruitment

Purposeful sampling was employed to recruit clinical consultants from public hospital trusts in South-Eastern Norway. We intentionally approached six hospital departments working with the treatment of medical conditions that could be related to patient self-responsibility. Considering the study’s time and budget constraints and the need to avoid long travel distances, we decided to approach hospitals in the southern part of Norway. Invitations to participate in the study were sent by email to the heads of departments. The heads of departments provided a list of clinicians, who were then invited to be included in the study. Two clinicians were identified by ‘snowball’ sampling. We wanted the sample to include at least three different specialities and to be adequate to achieve content validity. Since the study was designed as a conceptually driven thematic analysis (with pre-established categories), we aimed for saturation understood as populating ‘the pre-specified theoretical constructs with contextually relevant content’, rather than as ‘the emergence of new themes’, as is often the case in inductive analyses [38]. After coding the interviews, we concluded that all the pre-established constructs had been saturated (with several quotes for each category) and conducted no further interviews.

### Data collection

The interviews were conducted by the first author between May 2018 and February 2019. The topics addressed in the interviews included resource scarcity in healthcare, priority setting and the patient’s responsibility for their own health. Each interview lasted between 30 and 70 min. The face-to-face interviews took place at the participant’s workplace with the exception of one Skype interview. The interviews were audio-recorded with multiple devices and transcribed verbatim.

The semi-structured interview guide was developed by both authors and consisted of three vignettes and eight open-ended questions. A combination of vignettes and questions is seen as suitable to solicit discussion on potentially sensitive topics in individual interviews [39] since reasoning on hypothetical scenarios is perceived as less ‘threatening’ than being asked directly about one’s own experiences and practice [40]. The vignettes described hypothetical but realistic prioritisation scenarios and were used to gain insights into the clinicians’ own reasoning around attributions of personal health responsibility. The priority setting scenarios described in the vignettes varied with regard to the type and severity of the disease, level of resource scarcity and cost and complexity of treatment, but all touched upon contexts where appeals to personal health responsibility might become relevant (see Additional material). The first vignette presented to each clinician described a scenario of relevance for the clinician’s own field of expertise, while two others presented cases from other specialties.

Written informed consent to participate was obtained from all participants prior to the interviews. The participants were offered to review the illustrative quotes from their own interview before publication.

### Data analysis

The interview transcripts were analysed in NVivo 12. Analysis was undertaken thematically with the aid of a predetermined conceptual framework, which enabled conceptual clarification and differentiation. The origin of the conceptual framework can be found in the work on responsibility by Raz and Schicktanz [41] and Schicktanz and Schweda [42], who have approached responsibility as a ‘relational concept of moral agency’. We have revised, synthesised and developed the conceptual framework on dimensions supplying the work of Raz, Schicktanz and Schweda with the perspectives of Schmidt [43], Wikler [13], Feiring [6] and Bærøe and Cappelen [44]. The conceptual framework is reported on more extensively elsewhere by the authors [28].

According to this framework, appeals to personal health responsibility can be organised along four dimensions, each including specific subcategories (in parentheses), as follows: (i) level of conceptual abstraction (principle/criterion), (ii) temporality (prospective/retrospective), (iii) normative justificatory arguments (related to efficiency, fairness, desert or autonomy) and (iv) objections to responsibility (related to avoidability, causality, harshness, intrusiveness or inequity). The dimensions and the subcategories aided in sorting and analysing the data and enabled the comparison of empirical findings across interviews.

The thematic analysis used codes derived from the four dimensions of the framework and their subcategories. The pre-established codebook that guided the analysis is reported in Table 1. The coding procedure entailed processes of de-contextualisation and re-contextualisation [45]. First, relevant text sections were sorted into themes given by the four dimensions of the framework (Table 1, Column 1). Second, for each theme we identified text sections that fitted into the possible theme codes and categorised them accordingly (Table 1, Columns 2 and 3). This process de-contextualised the data from the original material. Third, the coding procedure was reviewed by the first author, and the quotes that were explicitly distinctive of each code were kept. To re-contextualise the material, we added context-specific descriptions to each code. Finally, the coding was discussed between the authors to obtain satisfactory reliability. Illustrative quotes were translated from Norwegian to English by the first author.

**Table 1:** Themes and codes applied to the qualitative thematic analysis in NVivo12

Theme label		Question asked to attribute code	Possible codes
Conceptualisation	Level of conceptual abstraction	*Does personal health responsibility refer to (i) substantial properties of the individual or (ii) is it framed as a general principle guiding policy?*	(i) Criterion (ii) Principle
	Temporality	*Is the action- event relationship in the attribution of personal health responsibility directed (i) forwards or (ii) backwards in time?*	(i) Prospective (ii) Retrospective
	Normative justificatory arguments	*Which normative arguments are used to justify personal health responsibility attributions?*	Efficiency-oriented Fairness-oriented Desert-oriented Autonomy-oriented
Application	Objections to responsibility	*Which arguments are used to oppose personal health responsibility attributions?*	Avoidability Causality Harshness Intrusiveness Inequity

## Results

Fifteen clinicians, six men and nine women, from three of the six departments approached agreed to be interviewed. The participants worked either at a centre specialised in morbid obesity and overweight treatment (*n* = 5), a department of infection medicine (*n* = 5), or a transplantation centre (n = 5). Table 2 summarises the participants’ backgrounds (age, speciality and country of education). Three of the approached departments (one mental health and two orthopaedic) did not agree to participate, one citing lack of time and staff as reason for refusal. The other two departments did not reply to our invitations to participate (reason for refusal unknown).

**Table 2:** Participants’ background information

Participant	Age	Medical specialty	Country of education
1	43	Neurology	Norway
2	45	Internal medicine	Norway
3	30	Registrar	Norway
4	56	Occupational medicine	Norway
5	35	Registrar (general practice)	Norway/Slovakia
6	55	Internal medicine (gastroenterology)	Norway
7	62	Internal medicine (gastroenterology)	Norway
8	58	Internal medicine (gastroenterology	Norway
9	47	Internal medicine (infectious diseases)	Norway
10	56	Internal medicine (infectious diseases)	Norway/Germany
11	37	Internal medicine (infectious diseases)	Hungary/Norway
12	45	Internal medicine (gastroenterology)	Norway
13	43	Internal medicine (gastroenterology)	Norway
14	45	Internal medicine (infectious diseases)	Germany, Norway, Austria, Italy
15	48	Internal medicine (infectious diseases)	Hungary

In the face-to-face interviews, the combination of open-ended questions and vignettes proved effective for eliciting both direct answers and in depth reflections, and data emerged in a balanced way from both approaches. However, the participants seemed more comfortable in expressing their own thoughts and practices when talking about their responses to the hypothetical scenarios of the vignettes.

The results from the thematic analysis of the 15 interviews are reported below and presented following the dimensions of the conceptual framework.

### Personal health responsibility: from general principle to specific criterion

All clinicians endorsed the view that the general population should be encouraged to take care of their health by adopting healthy lifestyles. Such an understanding viewed personal health responsibility as a principle of ‘responsible agency’.

*(People must) listen to the doctor’s advice, take care of themselves, take the medicines that we recommend, try as far as possible to stop smoking and lead a healthy lifestyle and all these things. So, people need to be made responsible because we can't do everything for them, we can at best give advice so that people can make choices in the best possible way and under the best conditions.* (Participant 12)

However, the possible introduction of a formal criterion for priority setting based on the individual’s responsibility for health was seen as more controversial. A formal criterion was seen to imply that individuals were ‘held responsible’ for their lifestyles and contribution to causing and improving their health condition in a way that had consequences for their treatment possibilities, timing and costs.

*(I) t is our job to inform and to educate about what is a healthy lifestyle. But I do not think that we can demand that they (the patients) have to live that way or they will not get treatment, or not be prioritised for treatment, or pay higher co-payments. I think that's wrong.* (Participant 10)

*Patients must take responsibility for their own health. I mean, it is very important, but I do not think that they ought to be punished if they do not manage to do it. I think those are two different things.* (Participant 12)

Despite the overall reluctance towards introducing personal health responsibility as a formal criterion, some clinicians endorsed self-responsibility as an informal criterion that might become useful in specific clinical priority setting situations.

*It should be an additional criterion, yes, but whether it should be a decisive criterion, that is another matter. But I think that patients should be better at taking care of their own health, absolutely.* (Participant 3)

### Retrospective and prospective understandings of personal health responsibility

Most clinicians recalled and discussed situations in which personal health responsibility had been used in clinical priority setting decisions. A retrospective understanding of responsibility played a role in two types of decision-making situations in which patients engaging in harmful behaviours were given lower priority – those where patients that had already been given treatment were ‘disqualified’ if they reengaged in harmful behaviours and those where high resource scarcity and inter-patient competition exist.

*When it comes to rehabilitation ( … ) it is quite expensive. So if you have already had one stay earlier, then you will not get it again ( … ) you get one chance.* (Participant 2)

*If a patient who has undergone lung transplantation begins to smoke again, and lung functionality is reduced as a result, then he or she should not be offered a new transplant. It is the same for the liver; if you have undergone liver transplantation due to alcoholic cirrhosis and start drinking again, and thus destroy your new liver, you will not be offered a new one. There is international agreement on this.* (Participant 7)

Further, the clinicians were familiar with the use of the responsibility criterion in a prospective sense. Here, patients were expected to ‘take responsibility’ for their future behaviour in order to access treatment. This responsibility attribution could require demonstrating motivation to adhere to the therapeutic program or implementing substantial changes to lifestyle.

*Everyone who comes to the first consultation is evaluated and then they are always told that ‘At consultation number two, you must have made a change’ - whether it is weight loss or smoking cessation.* (Participant 2)

*Patients sign a contract ( … ) (stating) that they will come to the appointments. At worst, they can be taken off the list (if they don't show up) and have to start all over again.* (Participant 3)

*I would never not offer treatment because of something that they have done in the past, (…) but if they cannot follow up, then one might have to take that into consideration for prioritisation.* (Participant 10)

*The requirement is that they must cooperate, they must show up to the appointments, and they must take the medicines that we recommend.* (Participant 12)

### Normative justificatory arguments: efficiency, fairness to others, desert and autonomy

The interviewed clincians used different arguments to decide when appeals to personal health responsibility seemed fair as a prioritisation criterion. It was common to justify the criterion with reference to the role of self-responsibility for the intervention’s effectiveness and for the optimal allocation of healthcare resources (i.e. efficiency-oriented normative arguments).

*We want to make sure that the patient is able to lose weight through lifestyle change, because surgery can only yield results through the patient's own lifestyle change.* (Participant 1)

*If the treatment effect is very strongly related to the drug being taken every day, then you need to know that your patient complies. So, when considering the treatment effect then you must consider compliance as well, as part of the picture.* (Participant 5)

*People with ongoing drug addiction are not fit for getting liver transplants because there is a probability that they will not be able to follow up (after the intervention). The chance that the liver would be wasted is high. Given that we have few livers, and that people die on the waiting list, we have to make such prioritisation decisions.* (Participant 8)

*If you are significantly overweight and you need a hip replacement, the risks related to the intervention are lower if you manage to lose weight first, and that is something you have a responsibility for yourself.* (Participant 11)

In the specific case of organ transplantation, some pointed out that patients’ unhealthy health behaviours might result in the ‘waste’ of potentially useful organs and that such ‘waste’ might undermine the sustainability of the organ supply system.

*If your 18-year-old son loses his life in a motorcycle accident (…) and then his liver goes to an elderly alcoholic – if this is known publicly, then the willingness to give organs will unfortunately be reduced, I think.* (Participant 6)

*It not only has to do with the specific patient, it is also about the reputation of the transplantation programme in society, because ( … ) if you were to repeatedly give new organs to a person with ongoing substance abuse, and that person destroys the organ again and again, what will happen to the donor numbers then? ( … ) We also have a responsibility to manage (the programme).* (Participant 13)

Moreover, we found some references to fairness to others, particularly when justifying attributions of personal health responsibility in settings of high resource scarcity.

*It has nothing to do with (the individual's ability to) benefit or culpability, but with my knowledge of the other patients who are in line (…)*. (Participant 1)

*If they (the patients) have received treatment earlier ( … ), then they have been given that opportunity and used their opportunity. Then they won't get that offer again, because there are so many others who can get it.* (Participant 4)

Marginal relevance was given to justifications based on desert. In such situations, personal health responsibility could be attributed based on the blameworthiness or praiseworthiness of the patient’s past conduct. Also, patients could be asked to show their effort, motivation and willingness to change, and their conscientiousness would be used to assess eligibility to receive treatment.

*They have to be able to eat regularly, they have to show that they can swap unhealthy for healthier foods, we have to see that they are willing to change.* (Participant 2)

*There is a lack of organs (…), and it is morally condemnable, when he or she chooses to drink again … so you could say that he or she was ill, an alcoholic … but still, we must expect that from the patient.* (Participant 7)

*(A patient with) alcoholic cirrhosis should be abstinent (from alcohol) for six months prior to transplant (...) Those who manage to be sober are motivated and understand the seriousness of the situation.* (Participant 9)

Overall, clinicians were reluctant to justify personal health responsibility with arguments unrelated to efficiency, such as those based on desert. The idea that people are responsible for their health and deserve to be prioritised accordingly, did not seem convincing to them.

*When we first have said that this patient is a good candidate for transplantation, then his or her background has nothing to say. We can't sit there like that and judge.* (Participant 7)

*There is no such thing as getting sick justly or unjustly.* (Participant 10)

*This person could have a good life anyway, right? Even though she uses drugs. If this drug user dies without a transplant and she could have gained health from a transplant, then it's probably not our job to say, ‘we can’t do it, because you're on drugs’.* (Participant 11)

Further, personal health responsibility could be justified with reference to the respect for patient empowerment and self-determination (autonomy-oriented justifications). Emphasising personal responsibility for one’s own health would signalise respect towards the patient, acknowledging their status as an autonomous person:

*The patients feel that they are given respect and that they are expected to (do something) ( … ) I think it can be beneficial.* (Participant 2)

*I have many patients that come and say ‘I don't get any help’. They get ‘pathologised’ in a way; they become completely helpless. I think: No, they must actually show that they can help themselves. With guidance.* (Participant 4)

*If we see that the patient has not made a real attempt at losing weight with diet and lifestyle change and such, then I think that should be tried first. We must have the patient on board. Patient involvement, not the least.* (Participant 12)

### Objections to responsibility attributions

All the five important objections to attributing personal health responsibility that were pre-specified as categories in the analytical framework were reflected in the data.

First, a common objection regarded the avoidability of the behaviour. Some clinicians pointed out that individual preferences and lifestyles are, to some extent, shaped by society and the environment we are born into. Thus, it would be unreasonable to claim that certain behaviours are avoidable or a matter of ‘good will’. They further thought that some cognitive and behavioural mechanisms are acquired throughout life, being largely influenced by our surroundings and lived experiences.

*We know it is very easy to choose the wrong things. One is very much bombarded by impulses in the wrong direction in today's society.* (Participant 1)

*(Morbid obesity) is a disease that involves lots of hormones, a lot of complex things in the brain (...). When it turns into morbid obesity (…), then I don't think it's about someone getting their act together*. (Participant 4)

*Obesity is probably not just an individual problem; it is multi-factorial, food accessibility, advertising ( … ). Making the individual responsible is a little unreasonable. The same goes for substance abuse, right? It's often people who come from disadvantaged contexts and poor upbringing and things have gone wrong in all possible ways, right?* (Participant 11)

*Well, it's like saying that one has become addicted to drugs ‘for pure fun’, right? (...). Substance abuse ( … ) is perhaps an expression of a very bad childhood, where the system has failed in the first place.* (Participant 15)

Second, some participants suggested the problem of identifying the extent to which the patient’s lifestyle is the actual cause of the health condition, given the multitude of factors affecting our health:

*It is very complicated to point exactly to the cause of the kidney failure; there are so many factors at play. So even if the patient has not listened to the doctor's advice (...), it is difficult to hold it against the patient.* (Participant 9)

*We don’t know what caused the overweight apart from the fact that the patient eats.* (Participant 10)

*To what degree has one exposed oneself to risk and to what degree is it an innate vulnerability (...). We do not have good enough systems, either, to distinguish what is primarily self-inflicted and not.* (Participant 13)

Third, holding patients responsible in a way that sanctions them for their behaviour was perceived by many doctors as disproportionate and harsh as it clashed with both a predisposition towards rescuing the sick and the duties of a public healthcare system, which should aid the sick.

*I cannot say that the patient should live with pain because he has played handball; I do not think the Norwegian public would agree with that (...). We treat heart attacks and lung diseases and very much of that is due to smoking, but patients are still treated for that.* (Participant 9)

*You can sit in your office (reading the patient journal) thinking ‘oh good lord’ - but then they come in, and you meet a human being. And then, well, it's hard to think about money* ( … ) *I think people have a responsibility for their own health, but when we have come so far that they have fallen ill, holding it against them is difficult because there is a person lying there who needs help.* (Participant 14)

Fourth, some pointed out that the application of personal health responsibility would be intrusive and jeopardise the fiduciary relationship between the clinician and the patient.

*I don't think requiring full abstinence is something that works in practice. It is very intrusive in a person's life.* (Participant 10)

*We are definitely interested in having a relationship based on trust with the patient. If one starts to deny patients effective care, it gets difficult to trust your doctor*. (Participant 14)

Finally, some objections regarded the inequity resulting in the concrete application of a criterion of personal health responsibility, since it would negatively affect the worst-off in society and challenge equal access to care.

*Many of our patients are people that are the most disadvantaged in society; they have experienced being bullied, abused ( … ). I don't mean that we should feel sorry for them all the way, but (…) it will be punishing them again and again.* (Participant 2)

*When it comes to this patient group, social inequalities are already a major burden, so financial sanctions in the form of higher co-payments will only create greater social inequalities ( … ). We would have a health system that contributes to increasing the social inequalities in health, and that is not what we want. We want to narrow the social inequalities in health.* (Participant 5)

*It would be like creating a (social) class distinction, which would destroy (our) nice model of a healthcare system (...). No, we cannot punish people for poor choices they have made in the past.* (Participant 15)

Others felt that personal health responsibility would be used to ‘hide’ moralistic reasoning or biases against the worst- off. This was clearly an uncomfortable issue to discuss.

*They will benefit equally from the procedure, and only one can get the liver: should it be the mother or the drug user? This is an ethical dilemma, but ultimately (the decision) should not be steered by the fact that one uses drugs and the other one not. In practice, however, it will probably affect (us), informally or unknowingly.* (Participant 11)

*People are full of burdens: one can smoke and drink and eat unhealthy (…) It's part of being human. If you are going to start to differentiate like ‘you have smoked, so you have to pay for your COPD medicines yourself’, that would not become a nice society, right?* (Participant 12)

## Discussion

In the following, we first reflect on some aspects of the findings, discussing them in light of the relevant literature, and then present some limitations to the study.

### Personal health responsibility – a contested criterion

Within the design of priority setting frameworks, the criterion of responsibility for own health has been highly controversial and categorised as one of the ‘contested criteria’ for resource allocation [4]. The clinicians in this study seemed to endorse personal health responsibility as a general principle for policy and conduct, prescribing to follow the doctors’ recommendations, show up to appointments and try to lead a healthy lifestyle. Yet, at the same time, clinicians were reluctant towards implementing a criterion based on this principle to the main priority setting framework and to the concretisation of this principle into policies of conditionality in access and prioritisation. This finding is in line with the stand taken in key Norwegian policy documents on healthcare prioritisation, which have pointed out that personal responsibility for health should only be considered in particular priority setting situations [28]. Similarly, a qualitative analysis of Norwegian healthcare stakeholders’ views on priority setting reported that most informants opposed personal responsibility as grounds for prioritisation [46]. A similar pattern has also been detected in the attitudes towards responsibility in the Norwegian general public, where only one third would support higher co-payments for self-inflicted illnesses [47].

Globally, individual responsibility for health, coupled with autonomy, figures as one of the cardinal principles in UNESCO’s Universal Declaration on Bioethics and Human Rights [48]. Generally, a principle of personal responsibility appears recurrently in relation to notions of ‘active citizenship’, encouraging and expecting individuals to take personal responsibility for, among other things, their health and lifestyle, becoming increasingly independent from welfare support [49–51]. Yet, as a principle of conditionality in access to care, self-responsibility for health does not seem to hold pivotal importance. A systematic review of citizens’ preferences for prioritisation criteria in healthcare concluded that concepts of responsibility for lifestyle and illness ‘may be relevant considerations in priority setting in some contexts, but some studies suggest them to be relatively minor considerations as compared with some other prioritisation criteria’ [52]. A recent study from a citizen forum in the Netherlands found disagreement among participants with regards to the role of individual lifestyle for decisions on the reimbursement of medical services. Here, the participants who favoured reimbursement pointed out that individuals often do not choose to behave in a unhealthy way (for instance because of substance addiction or for issues related to their social or family background) and in such case solidarity would be attributed more value, overriding the personal responsibility argument [53].

Five main objections to introducing personal health responsibility in health policies - avoidability, causation, harshness, intrusiveness, and inequity - have been widely discussed in the theoretical literature [5, 6, 44, 54–57]. These objections were all found to be relevant by our interviewees. Our empirical findings not only corroborate the moral relevance of such concerns, but also provide further insights into the concrete argumentations that clinicians in a public healthcare system use to support their views on personal health responsibility. The interviewed clinicians were most concerned about how using a personal health responsibility criterion would impact on the least advantaged, given the influence of external contingencies on the health-related behaviour of their patients. This finding is in line with the view that a strong emphasis on individual responsible agency, even without holding individuals accountable in a way that affects their right to healthcare, can be cause for concern from an ethical perspective. Such policies, suggesting that ‘individual behaviour change is achievable through empowerment and healthy choice*s*’ could result in unfairness towards the least advantaged by setting expectations that cannot be fulfilled by these individuals, because of the multitude of factors beyond their control that affect personal health-related behaviour and health [58]. Furthermore, patients from lower socioeconomic groups often have worse preconditions for being the ‘masters’ of their health and for taking on the role of “proactive patients”: responsible agency, as Elizabeth Anderson points out, ‘requires real options, awareness of those options, deliberative skills, and the self-respect needed to trust one’s own judgement’ [59].

Further, our interviewees feared that the use of a personal health responsibility criterion might hide ‘moralistic’ attitudes steered by prejudice towards specific ‘unwelcomed’ behaviours or societal groups. Our analysis identified that patients could be required to demonstrate their willingness and motivation to undergo treatment, showing some kind of ‘effort’ or own contribution. If the actual positive effect of such requirements on health outcomes is not supported by evidence, and if their implementation within policies of access is not transparent, these practices can be problematic. Evidence points to a link between pro-effort practices and moral appraisals. For instance, research in sociopsychology on attribution theory has suggested that moral judgements of obese individuals are often shaped by the assessment of their efforts towards weight loss [60–62]. Moralisation is a pertinent problem as health professionals’ moral appraisals of patients seem to increase in the context of time pressure and resource scarcity [63], both common traits of many priority setting situations in clinical contexts.

### The relevance of responsibility as an informal criterion in specific situations

The interviewees recalled specific clinical contexts where the patient’s self-responsibility assumed moral relevance, and many agreed that as an additional criterion, personal responsibility could be applied to prioritisation practices at the micro level. Retrospectively, personal health responsibility was found to be relevant when treatment had been previously provided, by disqualifying patients that reengaged in harmful behaviours. Also, in situations of high resource scarcity and inter-patient competition, some interviewees thought that it could be fair to give lower priority to patients that engage or have engaged in harmful behaviours. References to fairness to others were the main normative arguments of such retrospective attributions of responsibility. For instance, clinicians alluded to the fact that allocating more resources to patients who maintained detrimental health behaviours could be unfair to other patients, undermining *their* fair chance in a context of absolute resource scarcity.

Prospective personal health responsibility was attributed by setting expectations on what patients are required to do in order to access treatment, such as making positive adjustments to their own lifestyle. Most commonly, personal health responsibility was justified with reference to efficiency, viewing patient responsibility in relation to prognosis. We found that clinicians would consider the patient’s previous history of health-related behaviour to set expectations for future lifestyle to optimise treatment outcomes. Studies of other national public healthcare systems have also identified that attributions of personal responsibility acquire relevance when the aim is optimising the patients’ pre-operative health [17, 64]. There is a general consensus that prospect of success and medical utility are relevant criteria for the allocation of scarce and costly resources and that differences in patients’ expected health gains should figure into priority setting decisions [1]. Moreover, the Norwegian law states that the priority of an intervention increases with the health-benefit it yields [34]. In cases where pre- or post-operative lifestyle is a critical determinant of prognosis, there seems to be a(n) (efficiency-oriented) necessity for appealing to the patient’s responsibility. Such appeals to personal responsibility are generally difficult to dispute. Still, the risk for ‘value impregnation of factual aspects’, by which clinicians might (unconsciously) overstate the risks and health loss related to particular lifestyles based on personal values, is a relevant concern [65]. A systematic review of studies on the presence of implicit biases among healthcare professionals (i.e. unconscious associations that lead to negative evaluations of patients based on irrelevant characteristics) found correlations between the presence of such biases and lower levels of care [66]. Evidence suggests that implicit biases related to responsibility attributions can refer to several patient groups, including AIDS patients [67], brain-injured patients who have contributed to their injury [68, 69], injection drug users [15], self-harming mentally ill patients [70] and overweight patients [71–73].

Furthermore, empirical studies have shown that the real impact of pre-treatment lifestyle on intraoperative and post-treatment outcomes is not so clear. For instance, there is an increasing recognition that the six-month rule of abstinence imposed on alcoholic liver disease patients seeking liver transplantation is not a reliable predictor of the risk for relapse and might exclude candidates with otherwise favourable treatment outcomes [74, 75]. Similarly, injection drug users achieve treatment results from hepatitis-C therapy comparable to other patients, and abstinence requirements for this group could hardly be grounded on the maximisation of health gain [76]. On the other hand, smoking cessation before bariatric surgery has been shown to significantly lower the incidence of post-operative morbidity [77]. Although appeals to personal responsibility might be legitimately grounded in the concern for efficiency, such applications should be carefully evaluated and supported by evidence. The risk that considerations not relevant for determining benefit, cost-effectiveness and severity could play a role when prioritising patients on the waiting list is cause for concern and calls for greater attention from researchers and policy-makers.

### Limitations

One limitation deriving from having a conceptually driven qualitative design is the risk for selectivity in the use of the data. We guarded against this risk by a) constructing an analytical framework that captured a large range of themes based on the most important theoretical contributions on personal health responsibility found in the international literature and b) analysing the material systematically following the predefined themes and applying them throughout to all data by aid of a codebook (Table 1). Moreover, the authors documented any analytical choice separately to provide information on the interpretative process. We used the 15-point checklist suggested by Braun and Clarke [78] to ensure good practice in thematic analysis from transcription to report.

A second limitation concerns the sampling and the presence of non-response biases in the data collected, arising from the impossibility to interview the clinicians that refused to participate. It is not possible to assess whether non-participants shared common traits that would affect their views on personal health responsibility. However, we sampled our informants purposefully to represent clinicians working with different patient groups with lifestyle-related diseases. This variation allowed us to capture multiple perspectives. We reached data saturation when all the predefined themes were covered in several interviews. We assessed the gathered material as adequate to answer our research questions because the variation in responses shed light on different aspects related to personal responsibility attributions.

Moreover, there was some gender imbalance in our sample, having six males and nine females. Since women in general seem to be more reluctant to applying personal health responsibility as a criterion [47], this might have had impact on our results.

Given the interpretative efforts required to make sense of the data, an important element to assure analytical rigour was reflecting upon the role of the researchers and on the possible biases that our own position could bring into the analysis. In this regard, we adopted a strategy to minimise the risk for biases related to two issues. The first issue relates to the fact that the first author is a non-native Norwegian speaker. This could have limited the researcher’s ability to detect nuances in the language of the informants. We guarded against this risk by transcribing each interview verbatim and by collecting all relevant quotes in the original language. Further, the selection of quotes for publication was discussed with the second author (a native Norwegian). A second issue regards the fact that both authors have non-medical backgrounds. This might have influenced the importance given to certain themes. To avoid such biases, we used a systematic analytical approach that followed the predefined conceptual framework, which was based on the relevant theoretical literature. We captured and presented all themes of the theoretical framework, making sure that all interviews were given equal attention. Moreover, we emphasised the distinction between the presentation of the analytical findings and the in depth discussion of what we thought were the most relevant patterns.

We recognise that our findings might be limited to the specific egalitarian context of the Norwegian healthcare system, characterised by universal and tax-financed health services predominantly provided by the public sector. It might be the case that in systems with other ideological and cultural characteristics, where health services provision and funding are to a larger extent left to private actors, personal health responsibility might play a more important role in priority setting. Still, we believe that our findings can also be of interest in other healthcare settings facing rising healthcare demand and costs.

Finally, given that we studied only the clinicians’ reported attitudes and beliefs, multidisciplinary research is needed to investigate the scope and reach of the actual application of personal health responsibility as a criterion for eligibility and priority for healthcare in clinical situations.

## Conclusions

Our study provides important novel insights into the clinicians’ beliefs about personal health responsibility in priority setting in Norway, improving the empirical knowledge concerning clinicians’ views on the fairness and potential applications of personal health responsibility.

The combination of open-ended questions and vignettes allowed us to gain in depth knowledge on the different components that shape the understandings and discussion on personal health responsibility in clinical situations. Our study found that although personal health responsibility was perceived as difficult to implement within the main priority setting framework, there were clinical contexts where it could figure in prioritisation practices at the micro level. Moreover, retrospective and prospective attributions of personal responsibility in clinical contexts appeared to be intertwined and based on one another. This element has been given limited attention in the literature. Additional research on personal health responsibility would benefit from considering the multiple clinical encounters that shape doctor-patient relationships and that create the information basis for eligibility and prioritisation for treatment.

## Supplementary information

**Additional file 1.**

## Data Availability

The datasets used and analysed in the current study are available from the corresponding author upon reasonable request.

## Abbreviations

NCDs: Non-Communicable Diseases
RHAs: Regional Health Authorities
GP: General Practitioner
COPD: Chronic Obstructive Pulmonary Disease
UNESCO: United Nations Educational, Scientific and Cultural Organization
AIDS: Acquired Immune Deficiency Syndrome
