# Supplementary material for: ‘There is no such thing as getting sick justly or unjustly’ – a qualitative study of clinicians’ beliefs on the relevance of personal responsibility as a basis for health prioritisation
Source: BMC Health Serv Res. 2020 Jun 3;20:497. doi: 10.1186/s12913-020-05364-6 (PMC7268691; doi:10.1186/s12913-020-05364-6)
Supplement: Supplementary file 1 — Additional file 1. [file 12913_2020_5364_MOESM1_ESM.docx]

**Vignette 2**

Patient X (30 years) has a BMI of 37. The patient has tried to lose weight for many years without succeeding. The patient has type II diabetes, and being overweight negatively affects the patient’s quality of life. The patient investigates with the clinician whether it is possible to get help to lose weight through public healthcare. The clinician reports that there are two treatment options. Treatment option A (bariatric surgery) has good expected effect, moderate cost and moderate risk of complications. Treatment option B (weight-loss program at a rehabilitation centre) has moderate expected effect, and high cost, but involves low risk for the patient.

1) Which treatment option should be offered to the patient?

2) Should there be any requirements imposed on the patient to access treatment?

The patient is considered an adequate candidate for treatment option A, but must first succeed in losing some weight. The patient must also attend a healthy nutrition program. After six months, the patient has completed the program, but has not reached the weight-loss target.

3) Should the patient be offered treatment A anyway?

The patient undergoes bariatric surgery and loses weight accordingly, but does not succeed at maintaining the necessary lifestyle changes in the long term. The diabetic condition worsens and 30 years later the patient needs a kidney transplantation.

4) Should the patient be considered for kidney transplantation?

If so, should the patient be given lower priority than other patients or pay higher co-payments?

**Vignette 1**

Patient X (30 years) has been a heroin drug user for many years. The patient was infected with h the hepatitis C virus during adolescence, probably due to incautious use of syringes. There are two possible treatment options:

Treatment option A (drug therapy A) has a good expected effect (7 out of 10 achieve sustained virologic response) and moderate cost. However, the patient has to adhere to therapy for several months and there is a risk of significant side effects. Treatment option B (drug therapy B) has very good expected effect (9 out of 10 achieve sustained virologic response), but it is also very expensive. This therapy is shorter and has fewer side effects than A.

1) Which treatment option should be offered to the patient?

2) Should there be any requirements imposed on the patient to access treatment?

The patient is considered an adequate candidate for treatment option B, but is first required to be sober for six months. The patient does not succeed at this, despite the help offered by the healthcare system.

3) Should the patient be offered treatment B anyway?

The patient is offered treatment. However, the patient does not manage to adhere to therapy, and the chronic liver infection worsens, impacting liver functionality and connected organs. Twenty years later, the patient needs a new liver.

4) Should the patient be considered for liver transplantation?

If so, should the patient be given lower priority than other patients or pay higher co-payments?

**Vignette 3**

Patient X (30 years) has torn the cruciate ligament. The patient has been playing handball since childhood and has been injured repeatedly, in the ankle, meniscus and cruciate ligament. The patient has been operated on and has received physical therapy in the past. There are two treatment options for the new injury.

Treatment option A (surgery) has high expected effect, medium cost, and low risk. Treatment option B (physiotherapy) has less expected effect than A, very low risk and low cost.

1) Which treatment option should be offered to the patient?

2) Should there be any requirements imposed on the patient to access treatment?

The patient is considered as an adequate candidate for treatment option A, knee surgery. The doctor advises the patient to avoid activities that involve jumping, running and tackling due to the risk of injury. The patient explains that playing handball is very important for quality of life and that the patient cannot stop.

3) Should the patient be offered treatment A anyway?

After surgery, the patient continues to play handball. Thirty years later, the patient needs a knee prosthesis.

4) Should the patient be considered for knee replacement surgery?

If so, should the patient be given lower priority than other patients or pay higher co-payments?
